# Implications and Recommendations From a Project ECHO Series on Aging With IDD

**DOI:** 10.1093/geroni/igab046.1679

**Published:** 2021-12-17

**Authors:** Faith Helm, Jenni Mathews

**Affiliations:** 1 University of Rhode Island, Kingston, Rhode Island, United States; 2 Virginia Geriatric Education Center, Richmond, Virginia, United States

## Abstract

Ongoing evaluations of innovative educational programs provide opportunities for quality improvement. This paper reports results from a Project ECHO series on lifelong IDD and dementia. Participant responses (n = 85) were collected from spoke sites in various settings across the US. Using a 5-point (5 very effective) Likert scale, data were collected from assessment items on 4 didactic presentations and 5 case studies representing essential components of the ECHO model. Overall scores by spoke sites for satisfaction with the didactic and case presentations ranged from 3.94 to 4.94; relevance of case studies to the work setting ranged from 4.0 to 4.75. Knowledge gain questions showed consistently positive directionality. As a result of their participation, spokes rated intent to provide better care for patients (57% to 88%), train staff (62% to 81%), and educate family/caregivers (57% to 88%). Implications of findings for the application of quality improvement methods are discussed.

